# Impact of competitive anxiety on mood, sleep, and physical activity levels in young Tunisian karate athletes: A multidimensional prospective observational study

**DOI:** 10.1097/MD.0000000000048435

**Published:** 2026-05-01

**Authors:** Ahlem Belgacem, Atef Salem, Mevlüt Yildiz, Halil İbrahim Ceylan, Said Ben Hassen, Yassine Negra, Raul Ioan Muntean, Mohamed Jarraya

**Affiliations:** a High Institute of Sport and Physical Education of Sfax, University of Sfax, Sfax, Tunisia; b Research Laboratory: Education, Motricité, Sport et Santé (EM2S, LR19JS01), High Institute of Sport and Physical Education of Sfax, University of Sfax, Sfax, Tunisia; c Department of Training and Movement Science, Institute of Sport Science, Johannes Gutenberg-University Mainz, Mainz, Germany; d Coaching Sciences, Faculty of Sports Sciences, Mugla Sitki Kocman University, Mugla, Türkiye; e Physical Education and Sports Teaching Department, Faculty of Sports Sciences, Atatürk University, Erzurum, Türkiye; f Tunisian Research Laboratory Sports Performance Optimization, National Center of Medicine and Science in Sports (CNMSS), Tunis, Tunisia; g Higher Institute of Sport and Physical Education of Ksar Saïd, University of Manouba, Tunis, Tunisia; h Department of Physical Education and Sport, Faculty of Law and Social Sciences, University “1 Decembrie 1918” of Alba Iulia, Alba Iulia, Romania.

**Keywords:** competitive anxiety, karate, mood disturbance, physical activity, regression analysis, sleep quality, youth athletes

## Abstract

This study examined the impact of competition-related anxiety on psychological and behavioral parameters of Tunisian karate athletes. A total of 176 young Tunisian karate athletes (aged 16–25 years) with at least 1 year of training and competitive experience voluntarily participated in this study. Validated instruments were administered 1 week before and on the day of the competition, including the State-Trait Anxiety Inventory, the Pittsburgh Sleep Quality Index, the Brunel Mood Scale, and the International Physical Activity Questionnaire. Paired Wilcoxon signed-rank tests were used to compare changes over time, and multiple linear regression models were used to identify predictors of state and trait anxiety. Anxiety, mood, sleep, and physical activity changed from 1 week before to competition day (all *P* < .001). One week before, state anxiety was higher with tension (β = 1.22, *P* < .001), fatigue (β = 1.49, *P* < .001), and confusion (β = 1.14, *P* < .001); trait anxiety was higher with anger (β = 1.15, *P* < .001), depression (β = 1.38, *P* < .001), and fatigue (β = 0.42, *P* = .004), and lower with confusion (β = −0.36, *P* = .009). State anxiety was positively associated with sleep disturbances (β = 5.94, *P* < .001); trait anxiety with poorer subjective sleep quality (β = 2.87, *P* < .001) and longer sleep latency (β = 0.26, *P* < .001). Vigorous MET-minutes predicted lower state anxiety (β = −0.009, *P* = .02) and walking predicted lower trait anxiety (β = −0.007, *P* = .007). State anxiety increased with confusion (β = 1.47, *P* < .001) and decreased with vigor (β = −0.94, *P* = .003); trait anxiety increased with depression (β = 1.53, *P* < .001) and fatigue (β = 1.34, *P* < .001). Daytime dysfunction predicted higher state anxiety (β = 8.91, *P* < .001) and sleep latency predicted higher trait anxiety (β = 0.10, *P* = .031). Moderate MET-minutes predicted higher state anxiety (β = 0.008, *P* = .02). Increases in state anxiety aligned with higher tension (β = 0.93, *P* < .001), fatigue (β = 0.61, *P* = .004), and confusion (β = 1.89, *P* < .001), and decreases with depression (β = −0.54, *P* = .02). Increases in trait anxiety were driven by depression (β = 1.67, *P* < .001) and fatigue (β = 1.06, *P* < .001). Greater daytime dysfunction predicted increases in state anxiety (β = 9.28, *P* < .001), whereas longer sleep latency (β = 0.19, *P* < .001) and shorter sleep duration (β = −3.18, *P* < .001) predicted increases in trait anxiety; activity changes were not significant. Competition anxiety in young karate athletes is chiefly associated with mood disturbances and sleep parameters; implementing mood-regulation and sleep-focused strategies may reduce anxiety in youth combat sports.

## 1. Introduction

Anxiety is a common psychological hurdle for athletes, especially in combat sports. These high-pressure environments demand not only physical capabilities (strength, power, agility, endurance, and flexibility) but also strong mental resilience. Because of the direct physical confrontations involved, athletes often experience heightened stress, fear of injury, and performance anxiety before competitions.^[1]^ As a result, mental training becomes essential, incorporating techniques that support emotional control, behavioral flexibility, and interpersonal skills. These strategies help athletes manage stress effectively, thereby enhancing both performance and overall psychological health.^[2–4]^

Competitive anxiety is the tendency to perceive competitive situations as threatening, resulting in worry and tension.^[5]^ It often arises from a mismatch between external demands and an athlete’s perceived ability to cope with them. It manifests through cognitive symptoms, such as persistent self-doubt, and somatic symptoms, such as increased heart rate and muscle tension. While these reactions can impair performance, some athletes reinterpret them as indicators of readiness.^[6,7]^ The multidimensional model of anxiety^[5]^ divides this construct into cognitive anxiety (negative thoughts) and somatic anxiety (physiological arousal). Cognitive anxiety typically hinders performance, whereas somatic anxiety follows an inverted-U relationship, with moderate levels enhancing performance and extreme levels diminishing it. Hanin’s individual zones of optimal functioning further underscore that athletes’ interpretations of anxiety as facilitative or debilitative shape its impact.^[6,7]^ Collectively, these findings illustrate the complex interplay of cognitive, physiological, and behavioral factors in anxiety-performance dynamics.^[8,9]^

This perspective is grounded in a broader multidimensional framework for emotions, developed over decades of research. The model emphasizes the interconnected cognitive and physiological (somatic) components of emotional states and traits.^[10,11]^ Anxiety, as a primary focus within this framework, involves bidirectional interactions: cognitive appraisals can trigger physiological responses, which in turn influence subsequent cognitive processes.^[11]^ Although these components are linked, their activation depends on context, and their relationship to performance varies across tasks.^[7,9]^

Despite extensive research, gaps remain in understanding how anxiety interacts with other psychological variables in young athletes. Critical factors such as mood, sleep quality, and physical activity levels are vital for recovery, cognitive function, and stress regulation, yet are often overlooked in competitive settings. For example, optimal sleep supports cognitive performance; mood states, such as tension or vigor, directly influence emotional and physical readiness; and habitual physical activity can modulate pre-competition anxiety. Investigating these interconnected factors offers valuable insights into holistic anxiety management.

To our knowledge, this is the first study to investigate the effect of competition on multiple psychological parameters and the determinants of competitive anxiety in young karate athletes. The primary aim was to assess competition-induced anxiety, mood disturbance, and sleep quality. In addition, we examined relationships between anxiety and psychological parameters (sleep quality, mood state, and physical activity) to identify the most influential factor on competition-day anxiety. We hypothesized that relative to baseline, young karate athletes would show higher anxiety, greater mood disturbance, and poorer pre-competition sleep quality; competition-day anxiety would correlate positively with poorer sleep and negative mood, and negatively with habitual physical activity; and mood state, especially higher tension and lower vigor, would explain the most significant unique variance in competition-day anxiety compared with sleep quality and physical activity.

## 2. Materials and methods

### 2.1. Participants

One hundred and eighty athletes were initially enrolled in this study; who volunteered to participate and were recruited from multiple clubs across Tunisia; 4 were excluded from the final analyses due to incomplete questionnaires or loss to follow-up, resulting in a final sample of 176. The final sample included 91 males (52%) and 85 females (48%); mean age was 20.9 ± 2.8 years; we selected 16 to 25 years to focus on late adolescence and emerging adulthood, an age span in which emotion regulation, sleep patterns, and competitive exposure can change meaningfully. Restricting the sample to “young” athletes also reduces heterogeneity linked to older adult life-stage factors (e.g., full-time work, family responsibilities) that may influence sleep and anxiety. Inclusion criteria required at least 1 year of karate training and participation in at least 1 local or regional competition. Exclusion criteria included insufficient training or competitive experience, age outside the specified range, or lack of parental consent. This sampling strategy was intended to provide a representative cohort for evaluating competition-related anxiety and associated psychological factors in youth athletes. All athletes were enrolled only after appropriate consent had been obtained. Participants aged 18 to 25 years provided their own written informed consent, and those aged 16 to 17 years participated only after a parent supplied written informed consent and the athletes provided written assent. The study was conducted in accordance with the Declaration of Helsinki, and the protocol received full approval from the Ethical Committee for the Protection of Southern Persons (CPP SUD N° 0521/2023).

### 2.2. Experimental protocol

The first purpose (Study A) was to investigate the effect of competition on psychological parameters, including anxiety, sleep quality, mood state, and physical activity levels, among young Tunisian karate athletes. To this end, all participants completed a battery of questionnaires: the State-Trait Anxiety Inventory (STAI-Y)^[12]^ to measure anxiety, the Pittsburgh Sleep Quality Index (PSQI)^[13]^ to assess sleep quality, the Brunel Mood Scale (BRUMS)^[14]^ to evaluate mood state, and the International Physical Activity Questionnaire (IPAQ)^[15]^ to measure physical activity. These questionnaires were administered at 2 time-points, 1 week before the competition and on the day of the competition, to compare scores and identify changes associated with the event.

The second purpose (Study B) was to examine the influence of psychological parameters (sleep quality, mood state, and physical activity levels) alongside anxiety (state and trait) to determine which factors most strongly moderate anxiety on competition day. For this purpose, data from the same questionnaires (STAI-Y, PSQI, BRUMS, and IPAQ) at both time points were analyzed to evaluate the relationships between anxiety and the other variables.

### 2.3. Data collection and analysis

#### 2.3.1. The STAI-Y

The STAI-Y^[12]^ consists of 40 items divided into two 20-item multiple-choice subscales. The first subscale, the state anxiety (S-Anx), measures current feeling calm, tension, apprehension, nervousness, and worry – and includes items reflecting autonomic arousal. Items are rated on a 4-point Likert scale (1 = not at all, 2 = somewhat, 3 = moderately, 4 = very much). The second subscale measures trait anxiety (T-Anx) and addresses general feelings, with items indexing calmness, security, and confidence. Items are rated on a 4-point Likert scale (1 = almost never, 2 = sometimes, 3 = often, 4 = almost always). Subscale totals (range 20–80) are the sum of the 20 items, with higher scores indicating greater anxiety.

#### 2.3.2. PSQI

The PSQI was used to assess subjective sleep quality over the past month.^[13]^ The validated Arabic version was used in this study.^[16]^ The PSQI comprises 19 questions across 7 components: sleep duration, subjective sleep quality, sleep latency, sleep efficiency, sleep disturbances, daytime dysfunction, and use of sleep medication. The global score ranges from 0 to 21, with 0 indicating no problems and 21 indicating severe problems across components.

#### 2.3.3. BRUMS

The BRUMS^[14]^ comprises 32 items assessing mood states. The validated Arabic version was used.^[17]^ Items are grouped into 8 subscales: anger, tension, depression, vigor, fatigue, confusion, happiness, and calmness. Each item is rated on a 5-point Likert scale from 0 (not at all) to 4 (extremely). Participants indicate their current feelings and emotions. Subscale scores are calculated from 4 relevant items (range 0–16), providing a multidimensional assessment of emotional well-being.

#### 2.3.4. IPAQ

The IPAQ, translated into Swedish and then into English following the IPAQ manual to ensure reliability and validity,^[15]^ assesses 4 domains of physical activity: work, transportation, housework or gardening, and leisure. It also includes questions on sedentary behavior (time spent sitting). For each domain, the questionnaire records the number of days per week and the time per day spent in moderate (3–6 metabolic equivalent of task [MET]) and vigorous (>6 MET) activities. Walking time is also recorded for work, transportation, and leisure. Weekly physical activity in MET-minutes is calculated by multiplying the minutes spent in each activity category by the corresponding MET value.^[18]^ Two outcome measures were used: total MET-minutes per week and total minutes per week of moderate and vigorous activity.

The IPAQ was used because it is a widely applied, practical tool for field-based assessment of weekly physical activity in athlete samples. However, self-report is vulnerable to recall and social desirability bias, which may have introduced misclassification and attenuated true associations between physical activity and anxiety (bias toward the null). Future studies should complement questionnaires with objective monitoring (e.g., accelerometry/actigraphy or wearable-derived activity metrics).

### 2.4. Data analysis

Normality of continuous variables was examined using the Shapiro–Wilk test. Due to the non-normally distributed data, paired Wilcoxon signed-rank tests were used to compare anxiety, mood, sleep quality, and physical activity scores between 1 week before the competition and competition day. To characterize the interrelationships among psychosocial and behavioral factors and anxiety, we fitted separate multiple linear regression models at each time point, with S-Anx and T-Anx as dependent variables. Modeling multiple predictors jointly (mood states, sleep parameters, and physical activity) yields adjusted (independent) associations that account for shared variance among predictors and potential confounding, reduces the risk of spurious findings from numerous bivariate tests, and permits comparison of the relative contribution of each predictor (e.g., via standardized coefficients/partial *R*^2^). Correlations among explanatory variables were examined to identify potential collinearity, and correlation matrices were visualized to aid interpretation.

All tests were 2 sided with α = 0.05; exact *P* values are reported rather than thresholds, formatted as *P* = .xx for *P* ≥ .01, *P* = .xxx for .001 ≤ *P* < .01, *P* < .001 when smaller than .001, and *P* > .99 when exceeding .99.

Analyses were conducted in R version 4.4.0 using stats for normality testing, Wilcoxon tests, and linear models, ggpubr for visualization, and ggplot2 with ggcorrplot for correlation matrices.

## 3. Results

### 3.1. Impact of competition day on anxiety, mood states, sleep, and physical activity

**Figure 1.**
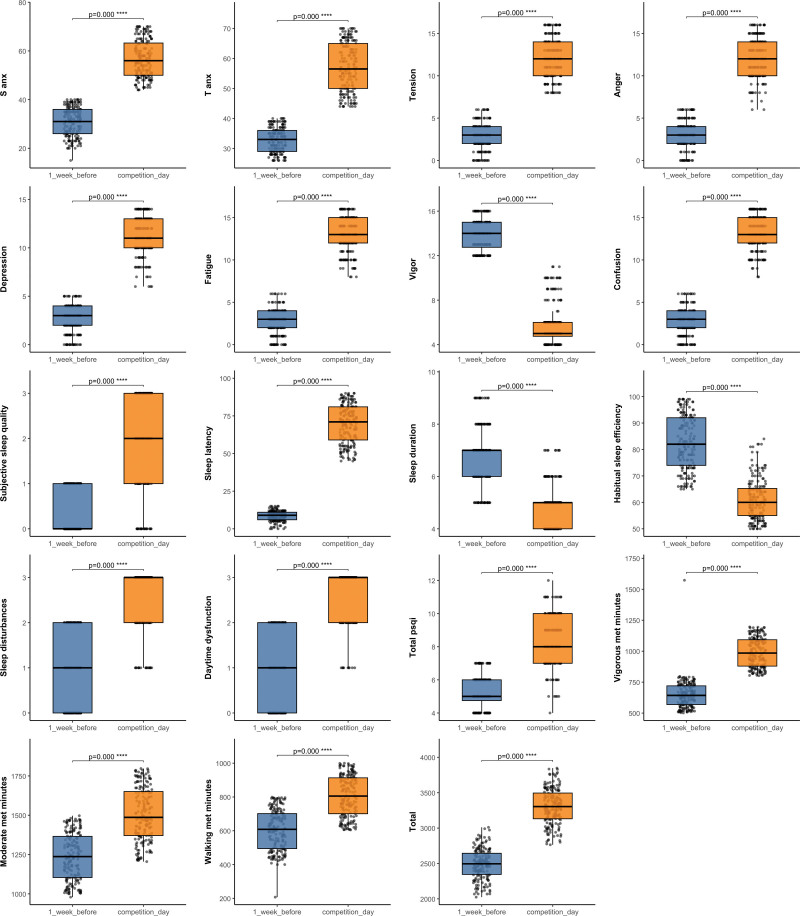
Anxiety, mood states, sleep, and physical activity 1 week before versus on competition day. PSQI = Pittsburgh Sleep Quality Index, S-Anx = state anxiety, T-Anx = trait anxiety.

There were significant differences between 1 week before competition and competition day across all variables of anxiety, mood states, PSQI, and IPAQ (*P* < .001; see Fig. 1).

### 3.2. Predictors of state and trait anxiety 1 week before the competition

Table 1 presents multiple linear regression estimates (β, SE, *P*) for the associations of mood states, sleep parameters, and physical activity with S-Anx and T-Anx 1 week before the competition, with model-fit statistics reported for each model.

**Table 1:** Multiple linear regression of mood, sleep, and physical activity on state and trait anxiety 1 week before competition.

Model	Predictor	Beta	SE	*P*	*R* ^2^	Adj. *R*^2^	RMSE	*F*	Model *P*
State anxiety									
Model 1 (mood states [BRUMS] → state anxiety [STAI-Y])	(Intercept)	17.526	2.702	.000	0.695	0.684	3.359	64.050	.000
	Tension	1.221	0.198	.000					
	Anger	0.095	0.189	.615					
	Depression	−0.359	0.216	.099					
	Fatigue	1.486	0.222	.000					
	Vigor	0.276	0.185	.138					
	Confusion	1.138	0.211	.000					
Model 2 (sleep quality [PSQI] → state anxiety [STAI-Y])	(Intercept)	28.470	5.025	.000	0.851	0.845	2.349	160.400	.000
	Subjective sleep quality	−0.088	0.707	.901					
	Sleep latency	0.028	0.057	.624					
	Sleep duration	−0.224	0.424	.599					
	Habitual sleep efficiency	−0.060	0.052	.246					
	Sleep disturbances	5.943	0.641	.000					
	Use of sleeping medications	–	–	–					
	Daytime dysfunction	–	–	–					
	Total PSQI	0.714	0.569	.211					
Model 3 (physical activity [IPAQ] → state anxiety [STAI-Y])	(Intercept)	32.725	5.258	.000	0.039	0.022	5.906	2.331	.076
	Vigorous MET-minutes	−0.009	0.004	.024					
	Moderate MET-minutes	0.003	0.003	.250					
	Walking MET-minutes	0.000	0.004	.970					
	Total	–	–	–					
Trait anxiety									
Model 1 (mood states [BRUMS] → trait anxiety [STAI-Y])	(Intercept)	25.481	1.745	.000	0.736	0.727	2.170	78.500	.000
	Tension	0.137	0.128	.286					
	Anger	1.148	0.122	.000					
	Depression	1.382	0.140	.000					
	Fatigue	0.424	0.143	.003					
	Vigor	−0.022	0.120	.854					
	Confusion	−0.362	0.136	.009					
Model 2 (sleep quality [PSQI] → trait anxiety [STAI-Y])	(Intercept)	45.683	5.014	.000	0.692	0.681	2.344	63.220	.000
	Subjective sleep quality	2.868	0.705	.000					
	Sleep latency	0.256	0.057	.000					
	Sleep duration	−0.573	0.423	.178					
	Habitual sleep efficiency	0.033	0.052	.520					
	Sleep disturbances	0.534	0.639	.405					
	Use of sleeping medications	–	–	–					
	Daytime dysfunction	–	–	–					
	Total PSQI	−3.004	0.567	.000					
Model 3 (physical activity [IPAQ] → trait anxiety [STAI-Y])	(Intercept)	40.836	3.633	.000	0.049	0.033	4.081	2.969	.033
	Vigorous MET-minutes	−0.004	0.003	.203					
	Moderate MET-minutes	−0.001	0.002	.602					
	Walking MET-minutes	−0.007	0.003	.007					
	Total	–	–	–					

Adj. *R*^2^ = adjusted *R*-squared, Beta (β) = regression coefficient, BRUMS = Brunel Mood Scale, *F* = *F*-statistic, IPAQ = International Physical Activity Questionnaire, MET = metabolic equivalent of task, *P* = *P* value, PSQI = Pittsburgh Sleep Quality Index, *R*^2^ = *R*-squared, RMSE = root mean squared error, SE = standard error, STAI-Y = State-Trait Anxiety Inventory (Form Y).

The multiple regression analysis model explored the influence of mood states (tension, anger, depression, fatigue, vigor, and confusion) on state anxiety 1 week before the competition, was significant (*F*(6, 169) = 64.05, *P* < .001), accounting for 69.46% of the variance in state anxiety (*R*^2^ = 0.6946, adjusted *R*^2^ = 0.6837). Predictors included tension (β = 1.2208, *P* < .001), fatigue (β = 1.4856, *P* < .001), and confusion (β = 1.1382, *P* < .001), all of which significantly and positively influenced state anxiety. Depression (β = –0.3593, *P* = .10) was negatively associated with state anxiety but was not statistically significant. Anger (β = 0.0955, *P* = .61) and vigor (β = 0.2761, *P* = .14) had no significant effect on state anxiety.

The analysis examining the relationship between mood states and trait anxiety 1 week before the competition showed a strong model fit (*F*(6, 169) = 78.5, *P* < .001), accounting for 73.59% of the variance in trait anxiety (*R*^2^ = 0.7359, adjusted *R*^2^ = 0.7266). Anger (β = 1.1475, *P* < .001), depression (β = 1.3822, *P* < .001), fatigue (β = 0.4239, *P* = .004), and confusion (β = –0.3616, *P* = .009) significantly predicted trait anxiety. Specifically, increases in anger and depression were associated with increases in trait anxiety, while fatigue was positively associated with trait anxiety, and confusion negatively predicted trait anxiety. Tension (β = 0.1371, *P* = .29) and vigor (β = –0.0221, *P* = .85) did not show any significant effects.

The multiple regression model, fitted to investigate the influence of PSQI variables on state anxiety, showed a strong fit (*F*(6, 169) = 160.4, *P* < .001), accounting for 85.06% of the variance in state anxiety (*R*^2^ = 0.8506, adjusted *R*^2^ = 0.8453). Significant predictors included sleep disturbances (β = 5.9432, *P* < .001), which positively influenced state anxiety. On the other hand, subjective sleep quality (β = –0.0878, *P* = .90), sleep latency (β = 0.0280, *P* = .62), sleep duration (β = –0.2237, *P* = .60), and habitual sleep efficiency (β = –0.0605, *P* = .25) did not significantly predict state anxiety. The total PSQI (β = 0.7141, *P* = .21) was also not a significant predictor.

The regression analysis for the relationship between trait anxiety and PSQI variables revealed a significant model fit (*F*(6, 169) = 63.22, *P* < .001), with an *R*^2^ of 0.6918 (adjusted *R*^2^ = 0.6808). Predictors included subjective sleep quality (β = 2.8683, *P* < .001) and sleep latency (β = 0.2560, *P* < .001), which positively influenced trait anxiety. Total PSQI (β = –3.0035, *P* < .001) had a significant adverse effect on trait anxiety. However, sleep duration (β = –0.5726, *P* = .18), habitual sleep efficiency (β = 0.0334, *P* = .52), and sleep disturbances (β = 0.5336, *P* = .41) were not significant predictors.

The model did not show a strong fit between physical activity and state anxiety (*F*(3, 172) = 2.331, *P* = .08), accounting for 3.91% of the variance in state anxiety (*R*^2^ = 0.0391, adjusted *R*^2^ = 0.0223). Vigorous MET-minutes (β = –0.0093, *P* = .02) significantly negatively predicted state anxiety. However, moderate MET-minutes (β = 0.0035, *P* = .25) and walking MET-minutes (β = –0.0001, *P* = .97) did not significantly influence state anxiety.

For trait anxiety, the physical activity model showed a significant fit (*F*(3, 172) = 2.969, *P* = .03), accounting for 4.92% of the variance in trait anxiety (*R*^2^ = 0.0492, adjusted *R*^2^ = 0.0327). Walking MET-minutes (β = –0.0071, *P* = .007) was a significant negative predictor of trait anxiety, indicating that greater walking activity was associated with lower trait anxiety. In contrast, vigorous MET-minutes (β = –0.0036, *P* = .20) and moderate MET-minutes (β = –0.0011, *P* = .60) did not significantly predict trait anxiety.

### 3.3. Correlation analyses between variables at 1 week before

**Figure 2.**
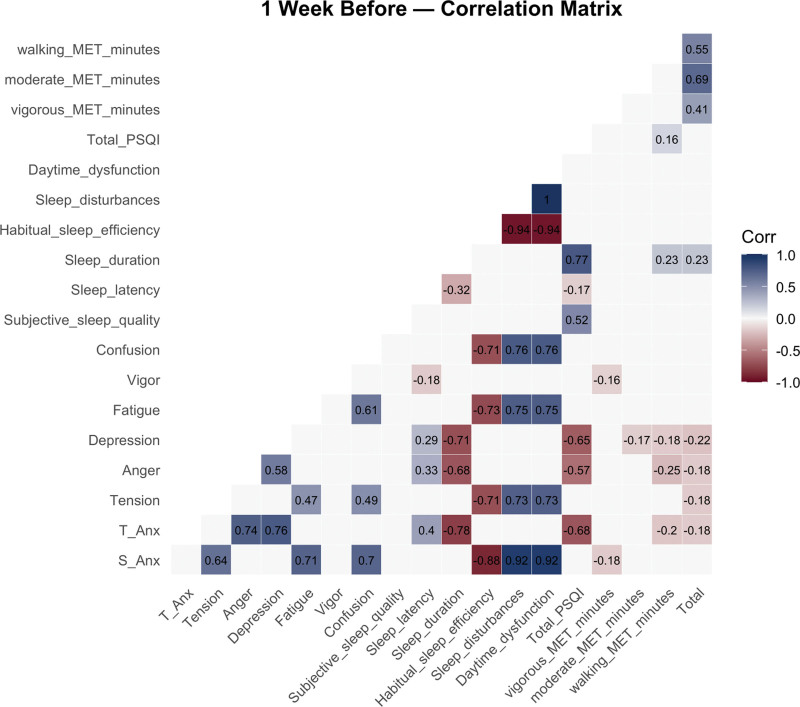
Correlation matrix 1 week before the competition. MET = metabolic equivalent of task, PSQI = Pittsburgh Sleep Quality Index, S-Anx = state anxiety, T-Anx = trait anxiety.

The correlation matrix of the relationships between anxiety, mood states, sleep quality, and physical activity 1 week before the competition is presented in Figure 2, and the detailed statistics for every pairwise correlation (*r* and corresponding *P*) are provided in Table S1, Supplemental Digital Content, https://links.lww.com/MD/R778.

The correlation analysis revealed that state anxiety reported significant, very strong positive correlations with fatigue (*r* = 0.73, *P* < .001), confusion (*r* = 0.72, *P* < .001), and tension (*r* = 0.66, *P* < .001). In addition, state anxiety showed a significantly very strong positive association with sleep disturbances (*r* = 0.94, *P* < .001). Trait anxiety, on the other hand, demonstrated significantly very strong positive correlations with depression (*r* = 0.76, *P* < .001) and anger (*r* = 0.75, *P* < .001). Furthermore, trait anxiety showed a strong negative correlation with sleep duration (*r* = –0.81, *P* < .001) and with total PSQI (*r* = –0.69, *P* < .001). Depression exhibited a significantly strong negative correlation with sleep duration (*r* = –0.75, *P* < .001) and a significantly strong negative correlation with total PSQI (*r* = –0.66, *P* < .001). Similarly, anger showed significant, very strong negative correlations with sleep duration (*r* = –0.70, *P* < .001) and significant, strong negative correlations with total PSQI (*r* = –0.57, *P* < .001). Fatigue was significantly and strongly associated with sleep disturbances (*r* = 0.77, *P* < .001) and daytime dysfunction (*r* = 0.77, *P* < .001). Habitual sleep efficiency demonstrated significantly very strong negative correlations with state anxiety (*r* = –0.88, *P* < .001) and fatigue (*r* = –0.74, *P* < .001). Sleep duration also revealed significantly very strong negative correlations with trait anxiety (*r* = –0.81, *P* < .001) and depression (*r* = –0.75, *P* < .001). Sleep disturbances and daytime dysfunction showed significant, very strong positive correlations with state anxiety (*r* = 0.94, *P* < .001), fatigue (*r* = 0.77, *P* < .001), and confusion (*r* = 0.76, *P* < .001). Finally, considering physical activity, the IPAQ score showed a significantly weak positive correlation with vigor (*r* = 0.03, *P* = .68) but significant weak negative correlations with depression (*r* = –0.25, *P* < .001) and anger (*r* = –0.21, *P* = .005). Vigorous activity exhibited a significantly moderate positive correlation with the IPAQ score (*r* = 0.45, *P* < .001), while moderate activity and walking demonstrated significantly strong positive correlations (*r* = 0.69 and *r* = 0.55, respectively, *P* < .001).

### 3.4. Predictors of state and trait anxiety on competition day

Table 2 presents multiple linear regression estimates (β, SE, *P*) for the associations of mood states, sleep parameters, and physical activity with S-Anx and T-Anx on competition day, alongside model-fit statistics.

**Table 2:** Multiple linear regression of mood, sleep, and physical activity on state and trait anxiety on competition day.

Model	Predictor	Beta	SE	*P*	*R* ^2^	Adj. *R*^2^	RMSE	*F*	Model *P*
State anxiety									
Model 1 (mood states [BRUMS] → state anxiety [STAI-Y])	(Intercept)	35.747	6.179	.000	0.507	0.489	5.377	28.910	.000
	Tension	0.325	0.213	.129					
	Anger	0.262	0.211	.216					
	Depression	−0.272	0.261	.299					
	Fatigue	0.245	0.256	.341					
	Vigor	−0.941	0.312	.003					
	Confusion	1.467	0.262	.000					
Model 2 (sleep quality [PSQI] → state anxiety [STAI-Y])	(Intercept)	33.444	11.136	.003	0.516	0.496	5.339	25.620	.000
	Subjective sleep quality	0.586	1.296	.652					
	Sleep latency	0.030	0.048	.540					
	Sleep duration	−0.005	1.344	.997					
	Habitual sleep efficiency	0.033	0.100	.740					
	Sleep disturbances	1.250	2.665	.640					
	Use of sleeping medications	–	–	–					
	Daytime dysfunction	8.915	1.557	.000					
	Total PSQI	−1.079	1.245	.387					
Model 3 (physical activity [IPAQ] → state anxiety [STAI-Y])	(Intercept)	42.983	7.524	.000	0.046	0.030	7.409	2.788	.042
	Vigorous MET-minutes	0.006	0.005	.172					
	Moderate MET-minutes	0.008	0.003	.018					
	Walking MET-minutes	−0.006	0.005	.230					
	Total	–	–	–					
Trait anxiety									
Model 1 (mood states [BRUMS] → trait anxiety [STAI-Y])	(Intercept)	14.867	7.062	.037	0.447	0.427	6.145	22.750	.000
	Tension	0.168	0.244	.492					
	Anger	−0.128	0.241	.596					
	Depression	1.527	0.299	.000					
	Fatigue	1.338	0.293	.000					
	Vigor	0.405	0.356	.258					
	Confusion	0.369	0.300	.220					
Model 2 (sleep quality [PSQI] → trait anxiety [STAI-Y])	(Intercept)	40.065	10.672	.000	0.619	0.603	5.117	38.940	.000
	Subjective sleep quality	−1.392	1.242	.264					
	Sleep latency	0.101	0.046	.031					
	Sleep duration	−1.024	1.288	.428					
	Habitual sleep efficiency	−0.167	0.095	.081					
	Sleep disturbances	4.727	2.554	.066					
	Use of sleeping medications	–	–	–					
	Daytime dysfunction	−0.607	1.492	.685					
	Total PSQI	2.051	1.193	.087					
Model 3 (physical activity [IPAQ] → trait anxiety [STAI-Y])	(Intercept)	53.562	8.233	.000	0.020	0.003	8.108	1.160	.327
	Vigorous MET-minutes	0.004	0.005	.500					
	Moderate MET-minutes	0.004	0.004	.260					
	Walking MET-minutes	−0.008	0.005	.145					
	Total	–	–	–					

Adj. *R*^2^ = adjusted *R*-squared, Beta (β) = regression coefficient, BRUMS = Brunel Mood Scale, *F* = *F*-statistic, IPAQ = International Physical Activity Questionnaire, MET = metabolic equivalent of task, *P* = *P* value, PSQI = Pittsburgh Sleep Quality Index, *R*^2^ = *R*-squared, RMSE = root mean squared error, SE = standard error, STAI-Y = State-Trait Anxiety Inventory (Form Y).

The multiple regression model relating mood states to state anxiety on competition day was statistically significant (*F*(6, 169) = 28.91, *P* < .001), explaining 50.65% of the variance (*R*^2^ = 0.5065, adjusted *R*^2^ = 0.4890). Vigor (β = –0.9413, *P* = .003) was a significant negative predictor and confusion (β = 1.4673, *P* < .001) was a significant positive predictor of state anxiety. Tension (β = 0.3253, *P* = .13), anger (β = 0.2621, *P* = .22), depression (β = –0.2724, *P* = .30), and fatigue (β = 0.2446, *P* = .34) were not significant.

For trait anxiety, the mood-state model was also significant (*F*(6, 169) = 22.75, *P* < .001), accounting for 44.68% of the variance (*R*^2^ = 0.4468, adjusted *R*^2^ = 0.4272). Depression (β = 1.5266, *P* < .001) and fatigue (β = 1.3376, *P* < .001) were significant positive predictors. Tension (β = 0.1678, *P* = .49), anger (β = –0.1282, *P* = .60), vigor (β = 0.4047, *P* = .26), and confusion (β = 0.3691, *P* = .22) were not significant.

Regarding sleep quality and state anxiety, the model was significant (*F*(7, 168) = 25.62, *P* < .001), explaining 51.63% of the variance (*R*^2^ = 0.5163, adjusted *R*^2^ = 0.4961). Daytime dysfunction (β = 8.9149, *P* < .001) was the only significant predictor, indicating greater daytime dysfunction was associated with higher state anxiety. Other sleep parameters were not significant: subjective sleep quality (β = 0.5858, *P* = .65), sleep latency (β = 0.0297, *P* = .54), sleep duration (β = –0.0052, *P* > .99), habitual sleep efficiency (β = 0.0331, *P* = .74), sleep disturbances (β = 1.2502, *P* = .64), and total PSQI score (β = –1.0794, *P* = .39). (Use of sleeping medications was excluded due to singularity.)

For sleep quality and trait anxiety, the model was significant (*F*(7, 168) = 38.94, *P* < .001), accounting for 61.87% of the variance (*R*^2^ = 0.6187, adjusted *R*^2^ = 0.6028). Sleep latency (β = 0.1009, *P* = .031) was the only significant predictor, with longer latency associated with higher trait anxiety. Other variables were not significant: subjective sleep quality (β = –1.3917, *P* = .26), sleep duration (β = –1.0243, *P* = .43), habitual sleep efficiency (β = –0.1674, *P* = .08), sleep disturbances (β = 4.7268, *P* = .07), daytime dysfunction (β = –0.6067, *P* = .68), and total PSQI score (β = 2.0511, *P* = .09). (Use of sleeping medications was excluded due to singularity.)

The model examining physical activity and state anxiety was statistically significant (*F*(3, 172) = 2.788, *P* = .04) but explained a small proportion of variance (*R*^2^ = 0.0464, adjusted *R*^2^ = 0.0297). Moderate MET-minutes (β = 0.0079, *P* = .02) showed a significant positive association with state anxiety, whereas vigorous MET-minutes (β = 0.0065, *P* = .17) and walking MET-minutes (β = –0.0057, *P* = .23) were not significant.

Finally, the model for physical activity and trait anxiety was not statistically significant (*F*(3, 172) = 1.16, *P* = .33), explaining 1.98% of the variance (*R*^2^ = 0.0198, adjusted *R*^2^ = 0.0027). None of the activity variables were significant predictors (vigorous MET-minutes: β = 0.0035, *P* = .50; moderate MET-minutes: β = 0.0041, *P* = .26; walking MET-minutes: β = –0.0076, *P* = .15).

### 3.5. Correlation analyses between variables on competition day

**Figure 3.**
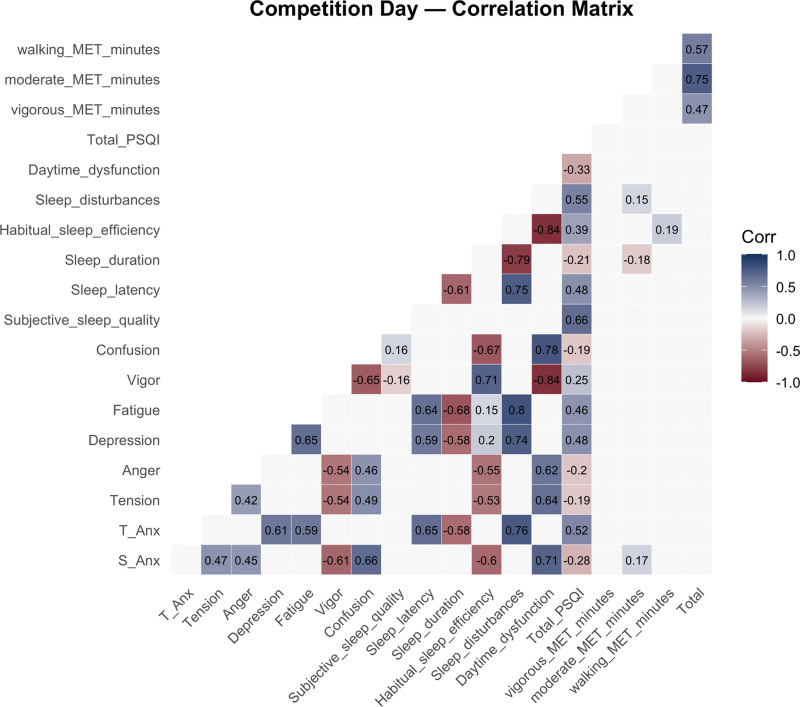
Correlation matrix the competition day. MET = metabolic equivalent of task, PSQI = Pittsburgh Sleep Quality Index, S-Anx = state anxiety, T-Anx = trait anxiety.

The correlation matrix of the relationships between anxiety, mood states, sleep quality, and physical activity on competition day was presented in Figure 3 and the detailed statistics for every pairwise correlation (*r* and corresponding *P*) are provided in Table S1, Supplemental Digital Content, https://links.lww.com/MD/R778.

The correlation analysis revealed that state anxiety reported significantly strong positive correlations with tension (*r* = 0.50, *P* < .001), moderate positive correlations with anger (*r* = 0.47, *P* < .001), strong positive correlations with confusion (*r* = 0.66, *P* < .001), very strong positive correlations with daytime dysfunction (*r* = 0.77, *P* < .001), and strong positive correlations with total PSQI score (*r* = 0.51, *P* < .001). In addition, state anxiety showed significantly strong negative correlations with vigor (*r* = –0.56, *P* < .001) and habitual sleep efficiency (*r* = –0.59, *P* < .001). Trait anxiety, on the other hand, demonstrated significantly strong positive correlations with depression (*r* = 0.58, *P* < .001), fatigue (*r* = 0.57, *P* < .001), sleep latency (*r* = 0.63, *P* < .001), and very strong positive correlations with sleep disturbances (*r* = 0.79, *P* < .001), while revealing a significantly strong negative correlation with sleep duration (*r* = –0.54, *P* < .001). Furthermore, tension exhibited moderate positive correlations with confusion (*r* = 0.46, *P* < .001) and strong positive correlations with daytime dysfunction (*r* = 0.67, *P* < .001). Similarly, vigor showed significant negative correlations with confusion (*r* = –0.54, *P* < .001) and very strong negative correlations with daytime dysfunction (*r* = –0.75, *P* < .001). Sleep disturbances demonstrated significant, very strong positive correlations with depression (*r* = 0.71, *P* < .001) and fatigue (*r* = 0.77, *P* < .001). Habitual sleep efficiency also revealed a significantly strong negative correlation with daytime dysfunction (*r* = –0.77, *P* < .001).

### 3.6. Drivers of changes in anxiety from 1 week before to competition day

Table 3 presents multiple linear regression estimates (β, SE, *P*) for the associations of mood states, sleep parameters, and physical activity with changes in anxiety (ΔS-Anx, ΔT-Anx; competition – 1 week), with corresponding model-fit statistics.

**Table 3:** Multiple linear regression of mood, sleep, and physical activity on Δ anxiety (competition – 1 week) for state and trait anxiety.

Model	Predictor	Beta	SE	*P*	*R* ^2^	Adj, *R*^2^	RMSE	*F*	Model *P*
State anxiety									
Model 1 (mood states [BRUMS] → state anxiety [STAI-Y])	(Intercept)	−5.845	3.286	.077	0.526	0.509	6.657	31.250	.000
	Tension	0.926	0.222	.000					
	Anger	0.177	0.215	.411					
	Depression	−0.539	0.227	.019					
	Fatigue	0.610	0.211	.004					
	Vigor	0.033	0.238	.891					
	Confusion	1.892	0.246	.000					
Model 2 (sleep quality [PSQI] → state anxiety [STAI-Y])	(Intercept)	7.622	2.982	.012	0.603	0.586	6.113	36.390	.000
	Subjective sleep quality	−0.378	0.990	.703					
	Sleep latency	0.067	0.052	.200					
	Sleep duration	−0.357	0.684	.603					
	Habitual sleep efficiency	0.025	0.082	.760					
	Sleep disturbances	−2.218	1.344	.101					
	Use of sleeping medications	–	–	–					
	Daytime dysfunction	9.278	1.430	.000					
	Total PSQI	0.462	0.853	.589					
Model 3 (physical activity [IPAQ] → state anxiety [STAI-Y])	(Intercept)	24.377	2.092	.000	0.010	−0.008	9.538	0.556	.645
	Vigorous MET-minutes	0.001	0.005	.819					
	Moderate MET-minutes	0.004	0.003	.209					
	Walking MET-minutes	0.000	0.004	.974					
	Total	–	–	–					
Trait anxiety									
Model 1 (mood states [BRUMS] → trait anxiety [STAI-Y])	(Intercept)	−1.162	3.525	.742	0.426	0.406	7.143	20.900	.000
	Tension	−0.088	0.238	.713					
	Anger	0.459	0.231	.048					
	Depression	1.670	0.244	.000					
	Fatigue	1.056	0.227	.000					
	Vigor	−0.097	0.256	.704					
	Confusion	−0.370	0.263	.161					
Model 2 (sleep quality [PSQI] → trait anxiety [STAI-Y])	(Intercept)	3.231	3.165	.309	0.529	0.510	6.488	26.970	.000
	Subjective sleep quality	−0.611	1.051	.562					
	Sleep latency	0.187	0.055	.001					
	Sleep duration	−3.176	0.726	.000					
	Habitual sleep efficiency	−0.098	0.087	.263					
	Sleep disturbances	1.360	1.427	.342					
	Use of sleeping medications	–	–	–					
	Daytime dysfunction	−1.857	1.518	.223					
	Total PSQI	1.194	0.906	.189					
Model 3 (physical activity [IPAQ] → trait anxiety [STAI-Y])	(Intercept)	24.873	2.044	.000	0.005	−0.012	9.321	0.286	.836
	Vigorous MET-minutes	−0.001	0.004	.775					
	Moderate MET-minutes	0.002	0.003	.590					
	Walking MET-minutes	−0.003	0.004	.437					
	Total	–	–	–					

Adj. *R*^2^ = adjusted *R*-squared, Beta (β) = regression coefficient, BRUMS = Brunel Mood Scale, *F* = *F*-statistic, IPAQ = International Physical Activity Questionnaire, MET = metabolic equivalent of task, *P* = *P* value, PSQI = Pittsburgh Sleep Quality Index, *R*^2^ = *R*-squared, RMSE = root mean squared error, SE = standard error, STAI-Y = State-Trait Anxiety Inventory (Form Y).

This model examined the influence of mood states (as measured by BRUMS scores) on changes in state anxiety. The multiple regression model was statistically significant (*F*(6, 169) = 31.25, *P* < .001), accounting for 52.59% of the variance in state anxiety (*R*^2^ = 0.5259, adjusted *R*^2^ = 0.5091). Significant predictors included tension (β = 0.9259, *P* < .001), fatigue (β = 0.6097, *P* = .004), and confusion (β = 1.8920, *P* < .001), all of which positively influenced state anxiety. Depression (β = –0.5388, *P* = .02) was negatively associated with state anxiety, while anger (β = 0.1773, *P* = .41) and vigor (β = 0.0326, *P* = .89) were not significant predictors.

The model, which evaluated the effect of mood states on the change in trait anxiety, was statistically significant (*F*(6, 169) = 20.9, *P* < .001), accounting for 42.59% of the variance (*R*^2^ = 0.4259, adjusted *R*^2^ = 0.4056). Depression (β = 1.6696, *P* < .001) and fatigue (β = 1.0558, *P* < .001) were significant positive predictors, while anger (β = 0.4591, *P* = .05) showed a weaker positive association. Tension (β = –0.0876, *P* = .71), vigor (β = –0.972, *P* = .70), and confusion (β = –0.3705, *P* = .16) did not significantly influence trait anxiety.

This model examined the relationship between sleep quality (as measured by PSQI components) and changes in state anxiety. The model was statistically significant (*F*(7, 168) = 36.39, *P* < .001), accounting for 60.26% of the variance (*R*^2^ = 0.6026, adjusted *R*^2^ = 0.586). Daytime dysfunction was the only significant predictor (β = 9.2777, *P* < .001). However, subjective sleep quality (β = –0.3781, *P* = .70), sleep latency (β = 0.0673, *P* = .20), sleep duration (β = –0.3566, *P* = .60), habitual sleep efficiency (β = 0.0252, *P* = .76), sleep disturbance (β = –2.2175, *P* = .10), and total PSQI score (β = 0.4616, *P* = .59), showing no significant effects.

The relationship between sleep quality and trait anxiety was assessed in this model. The model was statistically significant (*F*(7, 168) = 26.97, *P* < .001), accounting for 52.91% of the variance (*R*^2^ = 0.5291, adjusted *R*^2^ = 0.5095). Significant predictors included sleep latency (β = 0.1875, *P* < .001) and sleep duration (β = –3.1757, *P* < .001). However, subjective sleep quality (β = –0.611, *P* = .56), habitual sleep efficiency (β = –0.0981, *P* = .26), sleep disturbance (β = 1.3596, *P* = .34), daytime dysfunction (β = –1.857, *P* = .22), and total PSQI score (β = 1.1926, *P* = .19), were not significant.

The model investigating the effect of physical activity (MET per minutes) on state anxiety was not statistically significant (*F*(3, 172) = 0.5562, *P* = .64), with an *R*^2^ = 0.0096 (adjusted *R*^2^ = –0.0077). None of the predictors, including vigorous MET-minutes (β = 0.0011, *P* = .82), moderate MET-minutes (β = 0.0041, *P* = .21), and walking MET-minutes (β = –0.0001, *P* = .97), significantly influenced state anxiety.

Similarly, the model evaluating the influence of physical activity on trait anxiety was not statistically significant (*F*(3, 172) = 0.2859, *P* = .84), with an *R*^2^ = 0.005 (adjusted *R*^2^ = –0.0124). None of the predictors, including vigorous MET-minutes (β = –0.0013, *P* = .78), moderate MET-minutes (β = 0.0017, *P* = .59), and walking MET-minutes (β = –0.0032, *P* = .44), were significant.

### 3.7. Correlation analyses between the delta changes of variables

**Figure 4.**
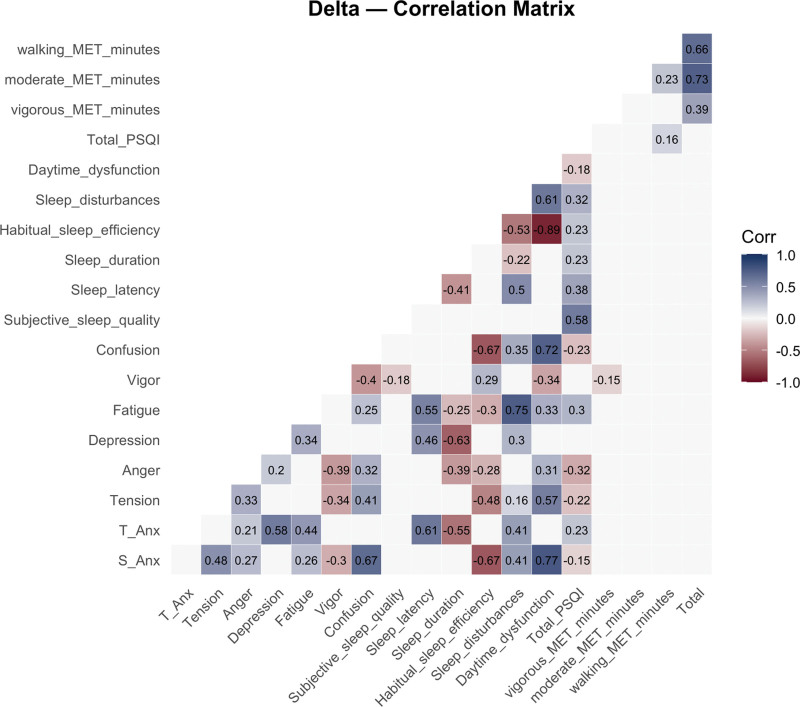
Correlation matrix of delta change. MET = metabolic equivalent of task, PSQI = Pittsburgh Sleep Quality Index, S-Anx = state anxiety, T-Anx = trait anxiety.

The correlation matrix for delta changes in variables provides insights into how shifts in various psychological, sleep, and physical activity factors interrelate, presented in Figure 4 and the detailed statistics for every pairwise correlation (*r* and corresponding *P*) are provided in Table S1, Supplemental Digital Content, https://links.lww.com/MD/R778.

The correlation analysis revealed that state anxiety reported significantly moderate positive correlations with tension (*r* = 0.43, *P* < .001), weak positive correlations with anger (*r* = 0.26, *P* < .01), strong positive correlations with confusion (*r* = 0.64, *P* < .001), moderate positive correlations with sleep disturbances (*r* = 0.41, *P* < .001), and very strong positive correlations with daytime dysfunction (*r* = 0.74, *P* < .001). In addition, state anxiety demonstrated a significantly strong negative correlation with habitual sleep efficiency (*r* = –0.65, *P* < .001). Trait anxiety, on the other hand, exhibited significantly strong positive correlations with depression (*r* = 0.57, *P* < .001), moderate positive correlations with fatigue (*r* = 0.46, *P* < .001), strong positive correlations with sleep latency (*r* = 0.61, *P* < .001), and moderate positive correlations with sleep disturbances (*r* = 0.40, *P* < .001). Furthermore, tension showed significantly weak positive correlations with anger (*r* = 0.32, *P* < .001), moderate positive correlations with confusion (*r* = 0.39, *P* < .001), and strong positive correlations with daytime dysfunction (*r* = 0.52, *P* < .001), while revealing a significantly moderate negative correlation with habitual sleep efficiency (*r* = –0.43, *P* < .001). Anger demonstrated significantly moderate negative correlations with vigor (*r* = –0.38, *P* < .001) and sleep duration (*r* = –0.38, *P* < .001). Depression was significantly negatively correlated with sleep duration (*r* = –0.62, *P* < .001). At the same time, fatigue exhibited significant positive correlations with sleep latency (*r* = 0.55, *P* < .001) and very strong positive correlations with sleep disturbances (*r* = 0.72, *P* < .001). Confusion revealed significant positive correlations with daytime dysfunction (*r* = 0.69, *P* < .001) and strong negative correlations with habitual sleep efficiency (*r* = –0.65, *P* < .001). Habitual sleep efficiency also demonstrated a significantly strong negative correlation with daytime dysfunction (*r* = –0.88, *P* < .001). Finally, considering physical activity, no significant correlations were observed between state/trait anxiety and activity levels. However, moderate and strong nonsignificant correlations were found between moderate MET-minutes and total MET-minutes (*r* = 0.73) and between walking MET-minutes and total MET-minutes (*r* = 0.66).

### 3.8. Summary of key regression findings

Across time-points, mood and sleep variables explained substantially more variance in anxiety than physical activity. One week before competition, higher state anxiety was independently associated with greater tension, fatigue, and confusion, and higher trait anxiety with anger, depression, and fatigue; sleep disturbances (state) and poorer perceived sleep quality and longer sleep latency (trait) were also relevant. On competition day, higher state anxiety was associated with higher confusion and lower vigor and was most strongly linked to daytime dysfunction, whereas higher trait anxiety was associated with higher depression and fatigue and longer sleep latency. In change (delta) models, increases in state anxiety were primarily driven by rising tension, fatigue, confusion, and daytime dysfunction, while increases in trait anxiety were driven by rising depression and fatigue together with longer sleep latency and shorter sleep duration; changes in physical activity were not significant predictors. Across models, mood and sleep variables accounted for substantially more explained variance than physical activity, which consistently contributed to small and inconsistent effects.

## 4. Discussion

This study aimed to investigate psychological and physiological factors that influence the anxiety levels of young karate athletes in competitive contexts. Specifically, we examined variations in state and trait anxiety, mood states, sleep quality, and physical activity 1 week before competition and on competition day. Regression analyses were conducted separately for each time point to identify moment-specific predictors, and for delta scores capturing change between the 2 assessments. The delta regression examined how changes in mood, sleep, and physical activity related to changes in anxiety, allowing us to detect dynamic linkages that may be missed when analyzing static time-points and to identify significant predictors of anxiety. We also examined correlations to characterize better relationships and changes among anxiety, mood, sleep, and physical activity over time.^[19]^ In a few places, the bivariate and multivariable results point in opposite directions. For example, confusion predicts lower trait anxiety after adjustment, even though it is generally part of a negative effect profile. Likewise, the total sleep score appears inversely related to trait anxiety once specific sleep facets such as poorer perceived sleep quality and longer time to fall asleep are in the model. In the change models, depression relates to decreases in state anxiety after controlling co-moving moods. These patterns are consistent with shared variance and statistical suppression among overlapping predictors, differences in scale polarity, and time-specific modeling. By design, the regression coefficients represent unique effects after removing common variance across mood and sleep, so an apparently paradoxical sign reflects the residual association rather than a contradiction of the simple correlations.

One week before the competition, state anxiety was positively correlated with tension and confusion, indicating a link between heightened anxiety and negative mood states, while vigor showed a negative correlation.^[20]^ Trait anxiety was associated with higher depression and fatigue, reflecting persistent emotional and physical stress.^[21]^ Poor sleep quality, particularly longer sleep latency and frequent disturbances, also emerged as significant correlates of trait anxiety.^[22,23]^ These findings suggest that mood and sleep disruptions are essential determinants of anxiety levels.^[24]^ On competition day, state anxiety was positively related to tension, anger, and confusion, highlighting mood disturbances as key factors.^[5,24]^ Vigor was inversely associated, consistent with reduced energy under stress.^[25]^ Trait anxiety remained linked to depression and fatigue, with stronger correlations to sleep disturbances and subjective sleep quality, underscoring the role of impaired sleep and mood dysregulation in exacerbating anxiety on competition day.^[26,27]^

Change analyses (delta) showed that shifts in state anxiety were strongly associated with increases in confusion and daytime dysfunction, alongside decreases in habitual sleep efficiency.^[22]^ Trait anxiety was significantly linked to longer sleep latency and higher depression levels.^[28]^ Poorer sleep quality, reflected in higher PSQI scores, correlated with reduced sleep duration and efficiency, as well as increased daytime dysfunction.^[22,29]^ Although physical activity metrics showed weaker associations, walking MET-minutes positively correlated with improvements in subjective sleep quality and duration. Overall, these findings highlight mood states and sleep quality as influential moderators of anxiety in competitive settings, with daytime dysfunction and sleep disruptions emerging as particularly important.^[26,27]^

Our analysis yielded several key findings. First, we observed significant differences in anxiety, mood, sleep quality, and physical activity between the 2 points. Anxiety levels, both state and trait, were notably higher on competition day than 1 week before, corroborating the common finding in sports psychology that anxiety rises as athletes approach competition.^[6,9]^ Mood states such as tension, anger, and confusion were also significantly elevated on competition day, while vigor, a positive mood state, decreased.^[16,30,31]^ This supports previous research linking competition-induced stress to mood disturbances such as frustration and irritability.^[32–34]^ Furthermore, sleep quality worsened on competition day, as athletes reported shorter sleep duration, more sleep disturbances, and greater daytime dysfunction.^[4]^ This is consistent with prior studies suggesting that stress and competition anxiety negatively affect athletes’ sleep patterns.^[22,33,34]^

Physical activity levels showed slight fluctuations across time-points, with moderate and vigorous activities more prevalent in the week preceding competition. This aligns with literature indicating that athletes tend to engage in higher-intensity training as they prepare for competition.^[35]^ However, despite the potential benefits of increased physical activity, it did not fully mitigate the negative psychological impacts of pre-competition anxiety and stress.^[20,25]^

The most striking difference between time-points was the increase in anxiety on competition day, particularly state anxiety, which aligns with the Yerkes–Dodson law.^[36]^ This law posits that moderate anxiety can enhance performance by increasing arousal and focus; however, when anxiety becomes excessively high, it can detract from performance and lead to adverse psychological and physiological effects.^[5,37]^ Our findings suggest that athletes experienced heightened anxiety due to the impending demands of competition, consistent with literature showing that anticipatory anxiety peaks closer to an event.^[38,39]^

We also observed significant increases in negative mood states (i.e., tension, anger, and confusion) on competition day, alongside a marked reduction in vigor. This is consistent with the competitive anxiety model, which holds that athletes often experience negative emotions, including irritability and frustration, in response to competitive stress and performance pressure.^[9,32]^ The decline in vigor suggests that athletes may feel physically and mentally drained as competition approaches, exacerbating stress and tension.^[40]^ This finding aligns with Vealey,^[41]^ who emphasized that heightened stress can lead to fatigue, decreased motivation, and lower energy levels, which can negatively impact performance.

The worsening of sleep quality on competition day was also evident. Athletes reported shorter sleep duration, more frequent sleep disturbances, and increased daytime dysfunction. Previous research has highlighted the negative effects of stress and pre-competition anxiety on sleep, with athletes often reporting poor sleep in the days leading up to competition.^[22,33]^ Stress-related arousal can interfere with the ability to relax, thereby reducing sleep quality.^[23,28]^ This suggests that, in high-pressure contexts, athletes may experience physiological responses that disrupt sleep, further contributing to increased anxiety.^[29]^

The regression models at each time point revealed significant predictors of both state and trait anxiety. At 1 week pre-competition, mood states such as tension, anger, and confusion were significant predictors of state anxiety. In contrast, sleep-related factors, including sleep disturbances and subjective sleep quality, were more strongly associated with trait anxiety.^[5,20]^ These findings are consistent with the cognitive–affective model of competitive anxiety, which suggests that mood changes, especially negative ones like anger or frustration, contribute to increased anxiety during competition.^[20]^

In our study, sleep disturbances, especially shortened sleep duration and increased sleep latency, emerged as significant predictors of trait anxiety, supporting research showing that poor sleep quality is strongly linked to heightened anxiety, particularly trait anxiety.^[28,42,43]^ Trait anxiety, which is chronic and enduring, appears especially vulnerable to the cumulative effects of poor sleep; inadequate rest heightens cognitive and emotional reactivity, which may amplify anxiety over time.^[44]^ These findings underscore the importance of managing sleep quality, particularly in the days leading up to competition, to reduce chronic anxiety.^[22]^

Mood states, including anger and confusion, were significantly correlated with state anxiety at both time-points, underscoring the acute emotional responses associated with competitive stress.^[29,38]^ This is consistent with findings that competitive anxiety can manifest immediately in response to negative mood states and perceived threats, which may be heightened during competition.^[38]^

Interestingly, our models showed that sleep disturbances were stronger predictors of trait anxiety than of state anxiety at both time-points. This is consistent with the idea that state anxiety is more responsive to immediate, situational factors (such as mood fluctuations and external stressors). In contrast, trait anxiety reflects a more enduring vulnerability influenced by longer-term factors such as sleep deprivation and chronic stress.^[45]^

When examining the delta models, we observed that changes in mood and sleep quality were significant predictors of changes in anxiety. For state anxiety, increases in tension and fatigue, along with decreases in vigor, were associated with greater increases in anxiety on competition day. This is consistent with the transactional model of stress,^[5]^ which suggests increasing stressors, such as competition-induced anxiety, elevating physiological and psychological strain, including tension and fatigue. The decline in vigor may reflect the draining effects of anticipating competition, leaving athletes mentally exhausted and less resilient to stress.^[9]^

For trait anxiety, the delta model showed that sleep disturbances and daytime dysfunction were strong predictors of change. This supports research indicating that disrupted sleep exacerbates vulnerability to anxiety, particularly when athletes are under high stress.^[34]^ Athletes who experience poor sleep in the days leading up to competition may become more susceptible to chronic anxiety, with lasting impacts on well-being.

Correlation analyses further revealed that mood states, especially tension, anger, and confusion, were strongly correlated with both state and trait anxiety. This aligns with the mental toughness framework, which emphasizes that athletes who can manage emotions and regulate stress are less likely to experience anxiety during competition.^[46]^ Our study supports the notion that emotional regulation plays a crucial role in managing anxiety in competitive contexts.

Sleep quality was also significantly correlated with both state and trait anxiety, consistent with the growing literature on sleep–anxiety relationships in athletes.^[22,26]^ Sleep disturbances, including reduced sleep quality and increased sleep latency, were strongly linked to increased anxiety, particularly trait anxiety, suggesting that poor sleep may contribute to long-term anxiety, especially under competitive stress.^[29]^

Physical activity levels, as measured by MET-minutes, showed weak correlations with anxiety. This suggests that, while physical activity may aid stress management and general well-being, its effect on competition-related anxiety may be less pronounced than that of mood and sleep-related factors.^[39]^ This aligns with research indicating that the relationship between physical activity and anxiety is complex and may depend on the intensity, frequency, and duration of exercise.^[21,43]^

To explain this relation between variables paradox, we clarify why directions sometimes differ between the simple correlations and the adjusted regression coefficients when predictors are interrelated and measured at different moments, which can yield sign changes due to multicollinearity and statistical suppression.^[47,48]^ Before competition, state anxiety aligns with strong simple associations for fatigue, confusion, tension, and sleep disturbances, and this carries through in adjusted models where those moods retain positive unique effects, consistent with the idea that specific facets can dominate aggregates in multivariable settings.^[49,50]^ The counterintuitive case arises for trait anxiety, where anger, depression, and fatigue remain positive as expected, yet confusion becomes negative after adjustment, a classic suppressor pattern in correlated predictors where the residualized portion of 1 mood relates differently once shared variance with other moods is removed.^[51,52]^ The same logic explains the sleep findings. Specific sleep facets such as poorer perceived sleep quality and longer time to fall asleep show positive links to trait anxiety, while the total sleep score turns negative once those dominant facets are included; because higher PSQI values indicate worse sleep, that negative coefficient does not imply protection but reflects overlap among PSQI components and the greater predictive weight of particular facets.^[13,22,44]^ In the change analyses, increases in tension, fatigue, and confusion track increases in state anxiety, while change in depression relates inversely once other moods are held constant, which is consistent with suppression among highly correlated affective states and with guidance on interpreting within-person change versus cross-sectional associations.^[53,54]^ For sleep, increases in daytime dysfunction emerge as the clearest driver of rising state anxiety over time, and longer time to fall asleep together with shorter sleep duration are most linked to rising trait anxiety, aligning with work showing tight coupling between insomnia symptoms, daytime impairment, and anxiety outcomes in athletes and active populations.^[26,42,44]^ Physical activity contributes small and context-dependent effects. Before competition, more vigorous activity relates to lower state anxiety and more walking relates to lower trait anxiety, whereas competition day and change models are largely null, a pattern that fits meta-analytic evidence that exercise effects on anxiety are modest and vary by dose, modality, and context.^[31,34]^ Overall, the correlations describe broad tendencies, while the adjusted coefficients describe unique, moment-specific or change-specific effects after shared mood and sleep variance is removed; under this lens, the sign reversals are statistically coherent and reinforce the central message that mood and sleep dominate the anxiety signal, with physical activity adding modest, situation-specific contributions.

Finally, this study has several strengths that increase confidence in the findings and their practical value for coaches. Data were collected in a real competitive context at 2 strategically timed points, 1 week before and on competition day, which enhances ecological validity and captures meaningful within-athlete change. The sample was relatively large for a single-sport youth cohort (n = 176) and was drawn from multiple clubs, improving representativeness. We used validated instruments, including culturally adapted versions when available (STAI-Y, PSQI, BRUMS, and IPAQ), and analyzed state and trait anxiety separately, along with mood, sleep, and physical activity. The analytic strategy matched the data structure, with nonparametric tests for non-normal distributions, prespecified models at each time point, and additional change models targeting delta scores. Correlation screening and visualization were used to check collinearity before regression, and the models explained substantial variance, indicating robust signals in predictors most relevant to practice, especially mood states and sleep components.

While this study provides valuable insights into factors influencing anxiety in karate athletes, several limitations should be acknowledged. First, the cross-sectional design limits causal inference; longitudinal designs with repeated assessments across training and competition phases would better clarify the directionality and temporal dynamics linking anxiety, mood, sleep, and physical activity, particularly in the days leading up to competition. Second, the sample size was relatively small and drawn from a specific population of young Tunisian karate athletes, which may restrict statistical power and limit generalizability; future research should include larger and more diverse samples across different competitive levels, combat sports (e.g., judo, taekwondo, boxing), and cultural contexts to test the robustness of these relationships. These relationships may vary with competition ruleset, weight-category demands, training periodization, and cultural context influencing sleep habits and anxiety appraisal. Third, the reliance on self-reported questionnaires for anxiety, mood, sleep, and physical activity may have introduced recall and social desirability bias; incorporating objective measures such as actigraphy for sleep, accelerometry for physical activity, and physiological indices of stress/anxiety (e.g., heart rate variability, salivary cortisol) could improve measurement precision and strengthen conclusions. Fourth, physical activity was assessed using a self-reported questionnaire rather than objective monitoring. This approach may overestimate activity levels and may not accurately capture short bouts, intensity distribution, or day-to-day fluctuations common in combat-sport training. Consequently, the observed associations between physical activity and competitive anxiety may be underestimated due to non-differential measurement error. Incorporating accelerometry/actigraphy and/or wearable training-load indicators (e.g., step count, session duration, heart rate-based training load) would strengthen future work by improving precision and enabling a clearer evaluation of whether activity changes meaningfully contribute to anxiety fluctuations near competition. Fifth, we did not conduct sex-stratified analyses or formally test sex-by-time interactions; therefore, potential sex-related differences in anxiety responses, mood fluctuations, or sleep disruption may have been missed and should be examined in adequately powered future studies. Finally, other psychological and social determinants, such as coping strategies, perceived stress, motivational climate, and social support; were not assessed and may also contribute meaningfully to competitive anxiety, warranting inclusion in future multidimensional models.

This study emphasizes the importance of monitoring psychological and behavioral indicators, specifically mood states and sleep quality, in the period preceding competition.

Coaches and support staff working with young karate athletes should prioritize early, routine detection of mood and sleep changes because increases in tension, fatigue, and confusion are strong predictors of heightened competitive anxiety, and sleep disruptions – especially disturbances, prolonged sleep latency, and daytime dysfunction – contribute to both immediate (state) and longer-term (trait) anxiety. In practice, this can be done with brief mood check-ins (e.g., short BRUMS subscales) and quick sleep check-ins during the week before competition and again on competition day to flag athletes who may need targeted support. When longer sleep latency or next-day dysfunction is evident, coaches should emphasize consistent sleep-focused routines (structured wind-down, limiting evening caffeine and screen exposure, relaxation breathing, and sleep hygiene). If tension and confusion rise as competition approaches, down-regulation strategies (diaphragmatic breathing, progressive muscle relaxation, mindfulness) combined with simple cue-based performance routines can reduce cognitive overload and restore attentional control; sport psychologists can reinforce these skills through cognitive restructuring and individualized preperformance routines to improve perceived control and help athletes reinterpret anxiety symptoms more adaptively. When fatigue increases and vigor declines, adjusting training load in the final week and prioritizing recovery (sleep extension, appropriately timed naps, and structured recovery sessions) may help protect readiness. Athletes showing persistently elevated trait anxiety alongside marked sleep disruption may benefit from referral to a qualified sport psychologist or clinician for individualized assessment and support. Overall, a multidisciplinary approach that blends physical preparation with tailored psychological and sleep support translates these predictors into low-burden, feasible steps that can be integrated into standard pre-competition routines to enhance well-being and competitive performance.

## 5. Conclusions

This study presents strong evidence that competitive stress significantly affects the psychological and physiological well-being of young karate athletes. Both state and trait anxiety increased on the day of competition, alongside higher negative mood states, particularly tension, anger, and confusion, and a marked drop in vigor. Sleep quality also declined, with longer sleep latency, greater daytime dysfunction, and lower sleep efficiency, which was associated with higher anxiety. Multiple regression analyses showed that mood states were the strongest predictors of immediate (state) anxiety. In contrast, sleep-related factors, especially disturbances and latency, played a larger role in explaining long-term (trait) anxiety. These findings highlight the importance of addressing both emotional and behavioral aspects of athlete preparation. Mood problems and poor sleep are not just symptoms of stress but active contributors to anxiety, and they therefore require targeted approaches. By identifying these key predictors, this study provides actionable insights for improving pre-competition readiness. Future research should build on these results using longer-term, experimental designs and objective physiological measures to clarify how psychological and behavioral factors interact to influence athletic performance under stress.

## Acknowledgments

We thank the participants for their participation in this study.

## Author contributions

**Conceptualization:** Ahlem Belgacem, Atef Salem, Mevlüt Yildiz, Halil İbrahim Ceylan, Said Ben Hassen.

**Investigation:** Ahlem Belgacem, Atef Salem.

**Methodology:** Ahlem Belgacem, Atef Salem, Mohamed Jarraya.

**Writing – original draft:** Ahlem Belgacem, Atef Salem, Halil İbrahim Ceylan.

**Writing – review & editing:** Ahlem Belgacem, Atef Salem, Mevlüt Yildiz, Halil İbrahim Ceylan, Said Ben Hassen, Yassine Negra, Raul Ioan Muntean, Mohamed Jarraya.

**Formal analysis:** Atef Salem.

**Supervision:** Mohamed Jarraya.

## Supplementary Material
