# Supplementary material for: Impact of competitive anxiety on mood, sleep, and physical activity levels in young Tunisian karate athletes: A multidimensional prospective observational study
Source: Medicine (Baltimore). 2026 May 1;105(18):e48435. doi: 10.1097/MD.0000000000048435 (PMC13138430; doi:10.1097/MD.0000000000048435)
Supplement: Supplementary file 1 [file medi-105-e48435-s001.pdf]

Correlation matrix of mood, sleep, and physical activity measures one week before competition and on competition day, and the delta change between one week before and the competition day. *Upper triangle shows Pearson correlation coefficients (r); lower triangle shows corresponding p-values. Variables: S_Anx = State anxiety; T_Anx = Trait anxiety; PSQI components include Subjective_sleep_quality, Sleep_latency, Sleep_duration, Habitual_sleep_efficiency, Sleep_disturbances, Daytime_dysfunction, and Total_PSQI; MET minutes are vigorous_MET_minutes, moderate_MET_minutes, and walking_MET_minutes. Negative r indicates an inverse association; positive r indicates a direct association.*

1 Week before the competition

	S_Anx	T_Anx	Tension	Anger	Depression	Fatigue	Vigor	Confusion	Subjective_sleep_quality	Sleep_latency	Sleep_duration	Habitual_sleep_efficiency	Sleep_disturbances	Daytime_dysfunction	Total_PSQI	vigorous_MET_minutes	moderate_MET_minutes	walking_MET_minutes	Total
S_Anx		-0,010	0,640	-0,040	-0,050	0,710	0,090	0,700	0,080	0,100	0,060	-0,880	0,920	0,920	0,120	-0,180	0,100	0,020	-0,010
T_Anx	0,943		0,090	0,740	0,760	0,100	-0,100	-0,050	-0,010	0,400	-0,780	-0,020	0,020	0,020	-0,680	-0,070	-0,050	-0,200	-0,180
Tension	0,001	0,251		-0,020	0,090	0,470	0,040	0,490	-0,010	0,090	-0,050	-0,710	0,730	0,730	-0,060	-0,060	-0,150	-0,090	-0,180
Anger	0,555	0,001	0,767		0,580	0,000	-0,080	-0,030	0,010	0,330	-0,680	0,030	-0,040	-0,040	-0,570	-0,060	-0,010	-0,250	-0,180
Depression	0,483	0,001	0,248	0,001		0,010	-0,070	-0,020	-0,020	0,290	-0,710	0,000	0,000	0,000	-0,650	-0,020	-0,170	-0,180	-0,220
Fatigue	0,001	0,184	0,001	0,953	0,899		-0,070	0,610	-0,050	0,060	0,020	-0,730	0,750	0,750	-0,030	-0,040	0,040	-0,030	-0,010
Vigor	0,247	0,175	0,595	0,279	0,366	0,343		0,110	-0,040	-0,180	0,020	-0,060	0,060	0,060	0,020	-0,160	0,130	0,070	0,040
Confusion	0,001	0,523	0,001	0,672	0,742	0,001	0,152		-0,070	-0,030	0,060	-0,710	0,760	0,760	0,000	-0,040	0,140	0,010	0,080
Subjective_sleep_quality	0,309	0,934	0,883	0,850	0,819	0,538	0,601	0,341		0,060	-0,040	-0,010	0,020	0,020	0,520	-0,070	-0,030	-0,040	-0,080
Sleep_latency	0,203	0,001	0,211	0,001	0,001	0,445	0,019	0,723	0,455		-0,320	-0,130	0,090	0,090	-0,170	-0,030	0,020	-0,060	-0,040
Sleep_duration	0,408	0,001	0,496	0,001	0,001	0,819	0,750	0,434	0,635	0,001		0,000	0,020	0,020	0,770	0,070	0,090	0,230	0,230
Habitual_sleep_efficiency	0,001	0,758	0,001	0,701	0,951	0,001	0,451	0,001	0,846	0,082	0,957		-0,940	-0,940	-0,040	0,100	-0,030	0,080	0,070
Sleep_disturbances	0,001	0,805	0,001	0,628	0,999	0,001	0,416	0,001	0,758	0,236	0,744	0,001		1,000	0,050	-0,090	0,050	-0,060	-0,050
Daytime_dysfunction	0,001	0,805	0,001	0,628	0,999	0,001	0,416	0,001	0,758	0,236	0,744	0,001	0,001		0,050	-0,090	0,050	-0,060	-0,050
Total_PSQI	0,116	0,001	0,446	0,001	0,001	0,700	0,813	0,976	0,001	0,025	0,001	0,631	0,544	0,544		-0,010	0,030	0,160	0,110
vigorous_MET_minutes	0,018	0,333	0,404	0,434	0,829	0,567	0,034	0,582	0,365	0,661	0,346	0,208	0,245	0,245	0,879		-0,070	-0,100	0,410
moderate_MET_minutes	0,193	0,546	0,050	0,871	0,025	0,614	0,086	0,069	0,737	0,782	0,211	0,668	0,531	0,531	0,659	0,349		0,070	0,690
walking_MET_minutes	0,798	0,009	0,236	0,001	0,018	0,736	0,385	0,911	0,599	0,422	0,002	0,292	0,411	0,411	0,034	0,206	0,378		0,550
Total	0,867	0,017	0,014	0,018	0,003	0,894	0,561	0,302	0,321	0,631	0,002	0,349	0,534	0,534	0,162	0,001	0,001	0,001	

Competition day

	S_Anx	T_Anx	Tension	Anger	Depression	Fatigue	Vigor	Confusion	Subjective_sleep_quality	Sleep_latency	Sleep_duration	Habitual_sleep_efficiency	Sleep_disturbances	Daytime_dysfunction	Total_PSQI	vigorous_MET_minutes	moderate_MET_minutes	walking_MET_minutes	Total
S_Anx		-0,061	0,472	0,449	-0,072	-0,013	-0,608	0,658	0,034	-0,028	-0,066	-0,600	-0,018	0,710	-0,285	0,102	0,167	-0,066	0,130
T_Anx	0,420		-0,051	-0,021	0,608	0,594	0,076	0,023	0,102	0,655	-0,581	0,099	0,764	-0,098	0,517	0,051	0,071	-0,100	0,025
Tension	0,001	0,505		0,419	-0,118	-0,099	-0,540	0,495	0,091	-0,083	0,098	-0,527	-0,090	0,643	-0,188	0,057	0,006	-0,054	0,005
Anger	0,001	0,782	0,001		-0,019	0,031	-0,542	0,461	0,105	-0,004	-0,061	-0,550	-0,023	0,618	-0,199	-0,019	0,063	-0,057	0,006
Depression	0,345	0,001	0,118	0,798		0,648	0,067	-0,029	0,055	0,589	-0,579	0,196	0,744	-0,135	0,479	0,031	0,047	-0,079	0,009
Fatigue	0,867	0,001	0,189	0,686	0,001		0,069	-0,021	0,032	0,641	-0,679	0,150	0,801	-0,114	0,455	0,050	0,122	-0,010	0,102
Vigor	0,001	0,314	0,001	0,001	0,375	0,364		-0,651	-0,156	0,056	0,028	0,712	0,059	-0,835	0,253	-0,039	-0,060	0,072	-0,025
Confusion	0,001	0,764	0,001	0,001	0,705	0,783	0,001		0,160	-0,017	-0,050	-0,670	0,021	0,776	-0,192	0,011	0,083	-0,119	0,005
Subjective_sleep_quality	0,650	0,178	0,232	0,165	0,468	0,671	0,039	0,034		0,076	0,021	-0,068	0,026	0,139	0,661	0,067	-0,002	0,143	0,099
Sleep_latency	0,715	0,001	0,274	0,958	0,001	0,001	0,463	0,828	0,313		-0,614	0,135	0,747	-0,100	0,478	0,083	0,031	0,011	0,066
Sleep_duration	0,384	0,001	0,194	0,424	0,001	0,001	0,713	0,510	0,786	0,001		-0,007	-0,791	0,007	-0,207	0,000	-0,185	0,048	-0,103
Habitual_sleep_efficiency	0,001	0,190	0,001	0,001	0,009	0,047	0,001	0,001	0,367	0,074	0,926		0,121	-0,841	0,393	-0,051	-0,068	0,194	0,022
Sleep_disturbances	0,815	0,001	0,236	0,763	0,001	0,001	0,438	0,783	0,731	0,001	0,001	0,111		-0,078	0,547	0,077	0,154	-0,062	0,112
Daytime_dysfunction	0,001	0,197	0,001	0,001	0,074	0,132	0,001	0,001	0,065	0,187	0,924	0,001	0,304		-0,326	0,076	0,132	-0,084	0,085
Total_PSQI	0,001	0,001	0,012	0,008	0,001	0,001	0,001	0,011	0,001	0,001	0,006	0,001	0,001	0,001		0,093	0,018	0,110	0,108
vigorous_MET_minutes	0,177	0,498	0,453	0,803	0,686	0,509	0,608	0,880	0,375	0,272	0,999	0,501	0,309	0,318	0,221		-0,002	-0,005	0,468
moderate_MET_minutes	0,027	0,352	0,936	0,409	0,538	0,106	0,427	0,271	0,977	0,682	0,014	0,369	0,041	0,081	0,814	0,979		0,140	0,745
walking_MET_minutes	0,385	0,188	0,473	0,454	0,299	0,900	0,341	0,117	0,058	0,885	0,529	0,010	0,416	0,266	0,146	0,953	0,064		0,570
Total	0,085	0,746	0,947	0,932	0,910	0,177	0,744	0,942	0,193	0,387	0,175	0,767	0,141	0,262	0,152	0,001	0,001	0,001	

Delta

	S_Anx	T_Anx	Tension	Anger	Depression	Fatigue	Vigor	Confusion	Subjective_sleep_quality	Sleep_latency	Sleep_duration	Habitual_sleep_efficiency	Sleep_disturbances	Daytime_dysfunction	Total_PSQI	vigorous_MET_minutes	moderate_MET_minutes	walking_MET_minutes	Total
S_Anx		-0,010	0,480	0,270	-0,110	0,260	-0,300	0,670	0,100	0,030	-0,040	-0,670	0,410	0,770	-0,150	0,010	0,100	0,020	0,080
T_Anx	0,919		-0,030	0,210	0,580	0,440	-0,060	0,000	0,120	0,610	-0,550	0,000	0,410	-0,020	0,230	-0,030	0,030	-0,050	-0,020
Tension	0,001	0,680		0,330	-0,060	0,030	-0,340	0,410	0,060	-0,090	-0,020	-0,480	0,160	0,570	-0,220	0,010	-0,070	-0,070	-0,070
Anger	0,001	0,005	0,001		0,200	0,070	-0,390	0,320	0,120	0,070	-0,390	-0,280	-0,030	0,310	-0,320	0,010	-0,030	-0,150	-0,090
Depression	0,129	0,001	0,403	0,009		0,340	-0,030	-0,050	0,050	0,460	-0,630	0,120	0,300	-0,090	0,080	0,030	-0,090	-0,100	-0,100
Fatigue	0,001	0,001	0,696	0,391	0,001		-0,060	0,250	0,000	0,550	-0,250	-0,300	0,750	0,330	0,300	-0,020	0,060	0,020	0,040
Vigor	0,001	0,426	0,001	0,001	0,721	0,445		-0,400	-0,180	-0,050	0,120	0,290	0,030	-0,340	0,130	-0,150	-0,020	0,120	-0,020
Confusion	0,001	0,955	0,001	0,001	0,475	0,001	0,001		0,040	0,000	-0,030	-0,670	0,350	0,720	-0,230	0,050	0,060	-0,040	0,040
Subjective_sleep_quality	0,206	0,102	0,393	0,124	0,548	0,970	0,014	0,644		0,100	-0,100	-0,040	0,020	0,080	0,580	0,050	0,040	0,120	0,110
Sleep_latency	0,719	0,001	0,217	0,340	0,001	0,001	0,514	0,984	0,198		-0,410	0,010	0,500	0,010	0,380	-0,010	0,050	0,040	0,050
Sleep_duration	0,582	0,001	0,831	0,001	0,001	0,001	0,117	0,720	0,203	0,001		0,030	-0,220	-0,030	0,230	0,030	-0,030	0,140	0,070
Habitual_sleep_efficiency	0,001	0,951	0,001	0,001	0,121	0,001	0,001	0,001	0,611	0,877	0,652		-0,530	-0,890	0,230	0,060	0,000	0,090	0,070
Sleep_disturbances	0,001	0,001	0,035	0,734	0,001	0,001	0,664	0,001	0,824	0,001	0,003	0,001		0,610	0,320	-0,020	0,060	-0,010	0,020
Daytime_dysfunction	0,001	0,843	0,001	0,001	0,230	0,001	0,001	0,001	0,267	0,944	0,662	0,001	0,001		-0,180	0,000	0,060	-0,020	0,030
Total_PSQI	0,046	0,002	0,004	0,001	0,275	0,001	0,083	0,002	0,001	0,001	0,002	0,002	0,001	0,020		0,050	0,010	0,160	0,110
vigorous_MET_minutes	0,919	0,738	0,876	0,882	0,651	0,755	0,043	0,520	0,533	0,864	0,701	0,426	0,839	0,986	0,512		-0,100	-0,010	0,390
moderate_MET_minutes	0,203	0,685	0,361	0,735	0,215	0,394	0,773	0,423	0,643	0,513	0,665	0,989	0,445	0,443	0,861	0,188		0,230	0,730
walking_MET_minutes	0,796	0,502	0,374	0,054	0,185	0,791	0,119	0,597	0,120	0,635	0,055	0,239	0,856	0,766	0,034	0,863	0,002		0,660
Total	0,307	0,818	0,323	0,254	0,197	0,578	0,749	0,579	0,164	0,550	0,384	0,327	0,750	0,717	0,133	0,001	0,001	0,001	
